# Unexpected Nephrotoxicity in Male Ablactated Rats Induced by *Cordyceps militaris*: The Involvement of Oxidative Changes

**DOI:** 10.1155/2013/786528

**Published:** 2013-02-24

**Authors:** Xiaowen Zhou, Yi Yao

**Affiliations:** Department of Pharmacy, Jiangsu Province Hospital of Traditional Chinese Medicine, Nanjing, Jiangsu 210036, China

## Abstract

Recently, many nutraceutical products containing the powdered or extracted parts of *C. militaris* have become available for health care. Due to the increased morbidity and mortality, poisonings associated with the use of herbs have raised the universal attention. Herein, we carried out the 28-day repeated toxicity test in male and female ablactated rats (three weeks old) given *C. militaris* powder orally at 0 (control), 1, 2, and 3 g/kg per day. Noticeable increments of serum aspartate and alanine aminotransferase (ALT and AST) levels were observed for both sexes, suggestive of weak hepatic toxicity. Nephrotoxicity characterized by tubular epithelium degeneration and necrosis was observed at the high dose, and the male rats were more susceptible to renal toxicity than female rats. In addition, the genes and protein expressions of novel markers of kidney toxicity, such as kidney injury molecule-1 (KIM-1) were enlarged in the renal cortex and the urine. Moreover, *C. militaris* treatment significantly decreased superoxide dismutase (SOD), catalase (CAT), and glutathione peroxidase (GPx) activities. However, the ratio of glutathione oxidized form (GSSG)/glutathione reduced form (GSH) was increased by *C. militaris* treatment. We conclude that dietary contamination with *C. militaris* may have renal toxicity potentials, at least in part by causing oxidative damage to the kidney.

## 1. Introduction

The Ascomycete genus *Cordyceps* includes over 500 species that are pathogens of arthropods. *Cordyceps* species, such as CCM and *C. sinensis*, are highly prized in traditional Chinese medicines, and they are also increasingly being studied and used in the West [1]. CCM extract, polysaccharides, and cordycepin have exhibited immunostimulatory and antitumor effects [2–4], and two dietary supplements related to cultured *Cordyceps* have been approved by the State Food and Drug Administration of China since 2002 [5, 6]. 

Herbal plants used in therapeutics or as dietary supplements date back beyond the recorded history. Conceptually, herbs are usually considered to be nontoxic by the general public due to their natural origin. Even though the expected medical effect is not achieved, their excessive intake is not regarded to be dangerous by the general public. However, renal pathology of plant medicines has emerged [7–9]. Considering the complexity of herbals, it is now necessary to evaluate their safety before the clinical use. CCM has been used in daily supplement, especially for the teenagers and the aged. Since children may be more sensitive than adults when given toxicant exposure [10], we performed a 28-day repeated toxicity study to investigate the toxicity of CCM on young ablactated rats, and obvious nephrotoxicity was unexpectedly found.

## 2. Materials and Methods

### 2.1. Animals and Treatments

Eighty-three-week-old SD rats (40 males and 40 females) weighing 80–90 g obtained from SLEK Lab Animal Center (Shanghai, China) were used in this study and acclimatized for one week in the standard animal house prior to administration. Room temperature was maintained at 22 ± 1°C, relative humidity at 55 ± 10%, and a 12 h light/dark cycle. Distilled water and food for rats were available* ad libitum*.

CCM was purchased from XuanzuPharma. co., Ltd. (Xinjiang, China) after confirming the morphology under microscopy. CCM powder was made by an ultrafine pulverizer. Powders were stored in a desiccator to protect against light and moisture. After vigorous stirring, CCM suspension containing distilled water as vehicle was given to rats by a syringe via gastrointestinal tract in a minute. Rats were randomly divided into four groups, each consisting of 10 males and 10 females, and given 0 (control), 1, 2, and 3 g/kg powered CCM once a day for 4 weeks. The animals were observed daily for clinical signs and mortality, and body weights were measured on the day of the administration (day 0) prior to treatment, 7, 14, 21, and 28 days after the start of administration. 

At the end, all animals were anesthetized with ether, weighed, and blood samples were collected from the abdominal aorta for haematology and biochemistry assays. Animals were then killed by exsanguination from the abdominal aorta. The tissues and organs, such as liver, spleen, kidneys, and testis (ovary), were fixed in 10% formalin for further histopathological diagnosis.

### 2.2. UPLC Conditions for the Identifications of Nucleosides and Nucleobases in CCM

Powered CCM samples were weighed into a volumetric flask. Approximately, 20 mL of water was added to the flask, which was subsequently sonicated for 1 h. After centrifugation for 10 minutes at 4000 ×g, sample solution was dried at 60°C, and the dried extract was dissolved in methanol-water (50 : 50, v/v). The sample was finally filtrated through a 0.45 *μ*M membrane filter prior to the analysis.

**Figure 1:**
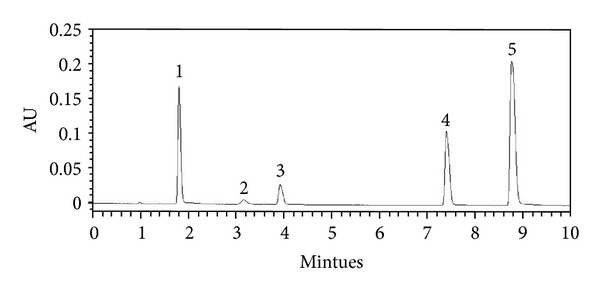
UPLC chromatogram of 5 standards. Peaks: 1, uridine; 2, adenine; 3, guanosine; 4, adenosine; 5, cordycepin.

**Figure 2:**
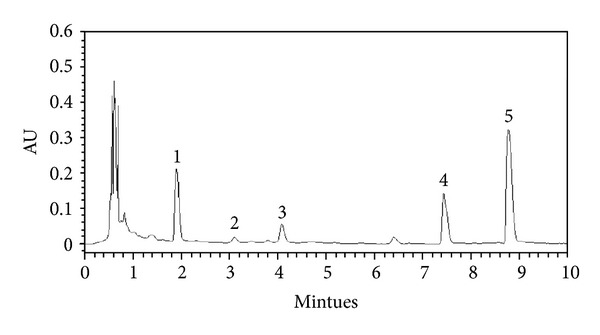
UPLC chromatograms of the water extracts from the fruiting bodies of *C. militaris*. Peaks: 1, uridine; 2, adenine; 3, guanosine; 4, adenosine; 5, cordycepin.

The LC system used for the method development and method validation consisted of a Waters ACQUITY UPLC (Milford, MA, USA) with PDA Detector. The chromatographic separation was performed on Waters ACQUITY UPLC BEH C18 column (100 mm × 2.1 mm, 1.7 *μ*m). Separation was carried out by linear gradient elution with methanol (2%, in 0–2 min; 2% −13%, in 2–10 min) and water (98% in 0–2 min; 98%-87% in 2–10 min) with a flow rate of 0.3 mL/min. The UV detection was operated at 260 nm, and the injection volume was 20 *μ*L. The external standard method was applied for the determinations. The identifications of nucleosides and nucleobases were attained by comparing their retention time and spectrum against known standards (Figures 1 and 2 and Table 1). 

### 2.3. Haematology and Biochemistry

Red blood cell count (RBC), white blood cell count (WBC), hemoglobin concentration, and white blood cell differential count (neutrophils, lymphocytes, monocyte, eosinophil, and basophil) were conducted using automated hematology cell counter CELL-DYN 3700 (Abbott Diagnostics, Santa Clara, CA, USA). Serum levels of aspartate aminotransferase (AST), alanine aminotransferase (ALT), total protein, albumin, blood urea nitrogen (BUN), creatinine, glucose, triglyceride, and total cholesterol (TC) were conducted using autoanalyzer (Type 7020, Hitachi, Japan). In addition, chloride (Cl), sodium (Na), and potassium (K) were measured using an ion autoanalyzer (Dri-Chem 800, Fuji, Japan).

### 2.4. Histopathological Examination

All histopathological tests were performed using standard laboratory procedures. The tissues were processed and trimmed, embedded in paraffin, sectioned to a thickness of 4–6 *μ*m, and placed onto glass slides. After stained with hematoxylin and eosin, the slides were observed, and the photos were taken using binocular Olympus DX45 microscope. 

### 2.5. Real-Time RT-PCR

Kidney tissues from renal cortex were harvested and quickly submerged in TRIzol solution (KeyGen Biotech. Co., Ltd., Nanjing, China). Total RNAs were extracted by using an RNeasy Mini Kit (Qiagen, Hilden, Germany). RNA concentration was assessed spectrophotometrically by measuring the A260/A280 absorbance ratios. The PCR primers were designed with Primer Express software (Applied Biosystems, CA, USA) and listed in Table 2. The amplification protocol consisted of 10 minutes at 95°C and followed by 36 cycles of amplification (95°C for 10 s, 54°C for 5 s, and 72°C for 15 s). Subsequently, the reaction was stopped at 95°C for 2 minutes, cooled (20°C for 1 minutes), and melted (70–94°C with plate readings set at 0.5°C) in an ABI 7300 real-time PCR system (Applied Biosystems) using the DNA-binding dye technique (SYBR Green). Differences in gene expression were calculated using cycle threshold (Ct) values, which were normalized against beta-actin of the same sample and expressed as relative transcript levels setting controls as 100%.

### 2.6. Western Blot

Kidney tissues from the renal cortex were loaded, and 30 *μ*g of protein from the supernatants was then separated by 10% SDS polyacrylamide gel electrophoresis and transferred onto nitrocellulose membranes. After blocking with 10% nonfat milk for 1 h at room temperature, the membranes were washed three times with PBST and incubated with antibodies against kidney injury molecular-1 (kIM-1) and GAPDH from Bioworld Biotech Co., Ltd. (Nanjing, China). Subsequently, membranes were incubated with peroxidase-conjugated secondary antibodies for 1 h at room temperature. Membranes were visualized with chemoluminescence reagents. Image pro plus (IPP) software for densitometry analysis is applied for the quantification of protein expressions. 

### 2.7. KIM-1 Assay in Urine

KIM-1 protein level was detected in the urine using an ELISA kit and according to the manufacturer's instructions (R&D Systems, Abingdon, UK). Urine was diluted per 10 or 100 to comply with the concentration intervals of the assay.

### 2.8. Oxidative Stress Markers in the Kidney

 Tissues from the renal cortex were harvested, and superoxide dismutase (SOD) and catalase (CAT) activities were determined according to recently described methods [11]. Glutathione oxidized form (GSSG) and glutathione reduced form (GSH) levels were analyzed according to methods [12] with a fluorescence microscope. Glutathione reductase (GR) activity was assayed using GSSG as a substrate and measuring the disappearance of NADPH [13]. The measurement of glutathione peroxidase (GPx) activity was performed according to a method based on the monitoring of the oxidation of NADPH at 340 nm. Glutathione S-transferase (GST) activity was examined as previously described [14]. 

### 2.9. Statistical Analyses

Data are presented as mean ± SD. Statistical analysis between groups was made by variance analysis (ANOVA), followed by Duncan's multiple range test. *P* < 0.05 was considered to be a significant difference. In addition, degrees of histopathological findings were subdivided into 4 degrees: (±) very slight; (+) slight; (++) moderate; (+++) severe. 

## 3. Result

### 3.1. Mortalities and Changes of Body Weights

No mortalities were recorded in all three different dosages tested. In the main study, diarrhea occurred sporadically during the treatment period in some males at middle and high doses. No alterations on body weight were observed as compared with the control group in all doses regardless of animal genders (Table 3).

### 3.2. Changes on the Organ-to-Body Weight Ratios and on the Clinical Indicators

After weighing the body and tissues, the coefficients of liver, kidneys, testis (ovary), and spleen to body weight were calculated as the ratio of tissues to body weight (Table 4). After exposure to 3 g/kg dose, significant increases of kidney-to-body weight ratio (*P* < 0.05) and testis-to-body weight ratio (*P* < 0.01) were found. 

No alterations were observed in triglyceride, glucose, albumin, and total protein levels between CCM-treated groups and the control group (Table 5), while rats at 3 g/kg dosage group had decreased total cholesterol serum concentrations in both genders (*P* < 0.05). Both 2 g/kg and 3 g/kg groups showed higher ALT and AST levels regardless of animal genders, respectively. 

### 3.3. Histopathological Findings

**Figure 3:**
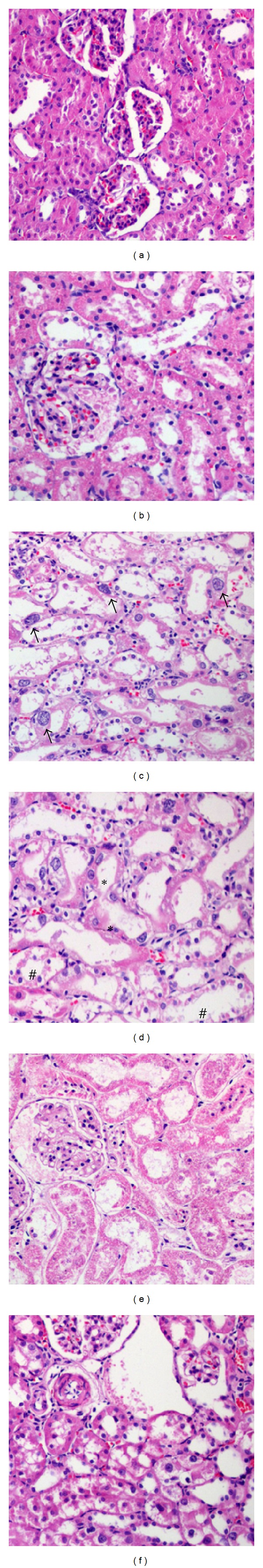
Micrographs showing histopathological changes in kidneys. (a) Normal glomeruli and renal tubules, the control group. (b) Proteinaceous effusion in the glomerular capsule as well as focal proteinaceous casts in the tubular lumina, male rats at 3 g/kg group. (c) Arrows indicate larger cells with one more nucleolus, male rats at 3 g/kg group. (d) Extensive necrosis and sloughing of proximal tubular epithelium. # indicates necrotic debris, and ∗ indicates tubular vacuolar degeneration, male rats at 3 g/kg group. (e) Dilated tubular epithelium without evidence of nuclear detail, male rats at 3 g/kg group. (f) Slight infiltration and moderate edema, female rats at 3 g/kg group.

Histological examination of haematoxylin and eosin stained sections displayed significant renal pathology at high-dose group compared to the control group (Figure 3(a)), whereas other tissues (brain, heart, liver, pancreas, testis, ovary, lung, and spleen) were unaffected. There was a lot of eosin stained proteinaceous effusion in the glomerular capsule as well as focal proteinaceous casts in the tubular lumina (Figure 3(b)). Besides, some renal tubular epithelial cells were significantly larger sized and characterized with one more nucleolus (Figure 3(c)). Cells have sloughed from the basement membranes in some tubules, and necrotic debris has accumulated in the lumina. Variably sized and clear vacuoles were also detectable in the cytoplasm (Figure 3(d)). Administration at 3 g/kg dose also induced necrosis of the *pars convoluta*, without evidence of nuclear detail (Figure 3(e)). The above-mentioned lesions were significantly attenuated in the female compared with the male, which means the nephrotoxicity was specific for the male (Figure 3(f) and Table 6). 

### 3.4. KIM-1 Expression

**Figure 4:**
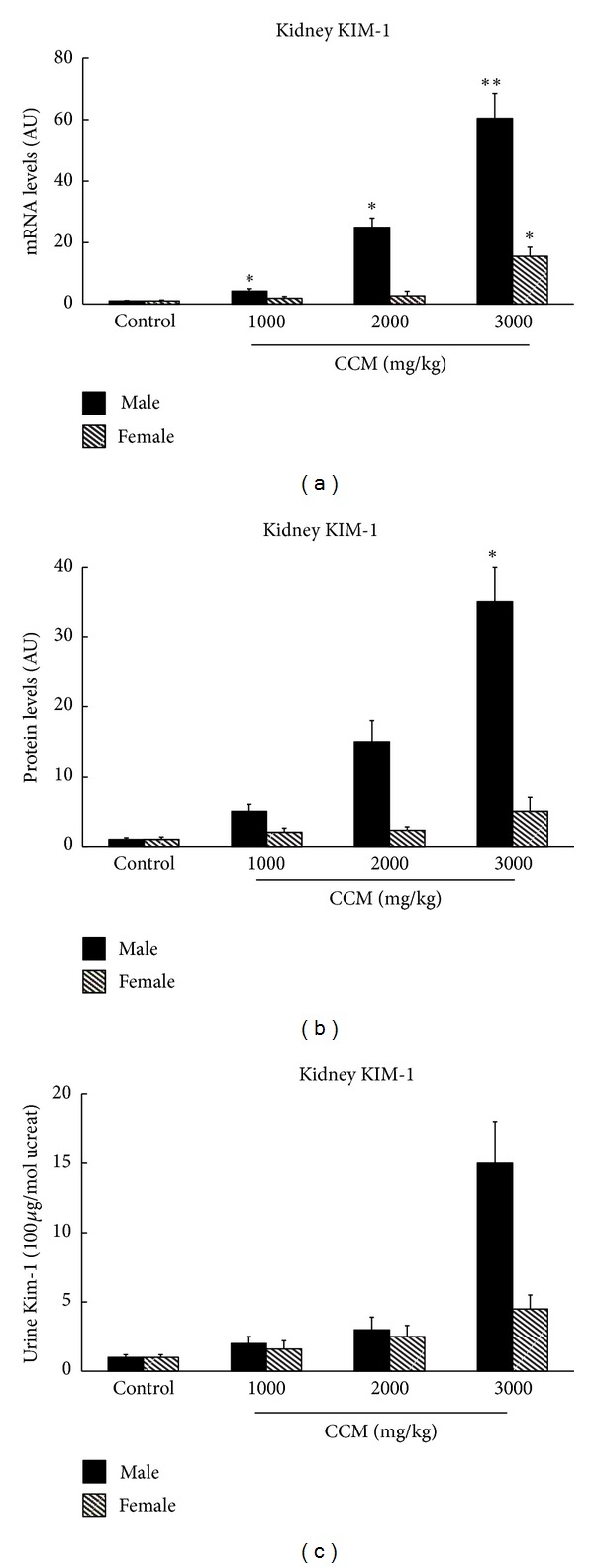
KIM-1 gene expression (a) and protein level (b) in renal cortex of kidney and in urine (c) from rats orally given CCM for 28 days. Urines were collected for 24 h via metabolism cage and blood and kidney were collected. The cortex section of kidney was separated (*n* = 10 for each group). The results are expressed as a ratio to the housekeeping gene mRNA levels, AU, arbitrary unit. *P < 0.05.

CCM treatment caused significant up-regulation of kIM-1 gene and protein expression in the renal cortex and the urine (Figure 4). In details, kIM-1 mRNA levels in male rats were increased of 4-, 20-, or 61-fold versus control by 1, 2, and 3 g/kg treatment, respectively (Figure 4(a)), whereas kIM-1 mRNA increment in female rats was only observed at the high dose (16-fold versus control). The protein level of kIM-1 also amplified significantly in male rats (35-fold) (Figure 4(b)). The same tendency, although not statistically significant, was noted in urine kIM-1 expression in male rats (Figure 4(c)). Nevertheless, no significant difference of kIM-1 protein level was observed in both renal cortex and urine for the female rats (Figures 4(b) and 4(c)). 

### 3.5. Antioxidant Enzyme Activities

Antioxidant enzyme activities (SOD, CAT, GR, GPx, and GST) and the oxidative stress maker GSSG/GSH ratio in renal cortex of ablactated rats given CCM are summarized in Table 7. For male rats, a significant reduction in SOD, CAT and GPx activities in 3 g/kg group was observed compared with the control group. The same tendency, although not statistically significant, was noted in GR activity. As expected, GSSG/GSH ratio was enhanced in CCM-treated rats. No changes were observed in the GST activity. For female rats, only SOD activity exhibited a decline and GSSG/GSH ratio showed an apparent increase, and the differences reached the level of statistical significance.

## 4. Discussion

It has been generally accepted that *Cordyceps* species have no critical toxicity for major organs [15]. Some commercial products are available in the market nowadays such as didanosine from *C. militaris* [16]. The nephrotoxicity characterized by necrotic tubules observed at 3 g/kg group in our study was completely unexpected. Kidney changes were prominent in high-dose group (only males) and featured proteinaceous effusion in the glomerular capsule and multiple tubular lesions, including degeneration, necrosis, and dilation.

Nephrotoxicity is a major complication characterized by morphological destruction of intracellular organelles and cellular necrosis and followed by functional alterations including the depletion of the antioxidant defense system and mitochondrial damage [17]. Oxidative damage is thought to be one of the main mechanisms involved in nearly all chronic renal pathologies [18]. In order to explore this possibility, we assessed the activity of renal antioxidant enzymes SOD, GR, GPx, GST, and CAT. In detail, SOD and CAT are the main antioxidant enzymes in the body, which scavenge unwanted O_2_, H_2_O_2_, and ROOH produced by free radical. SOD catalyzes superoxide radical dismutation, and CAT further removed hydrogen peroxide. We observed a significant fall in the activities of kidneys SOD and CAT in the rats fed with CCM. GSH is an important nonenzymatic antioxidant system in kidney. It can act as a neutralizer of oxidant metabolites in a reaction catalyzed by the GST enzyme, or as a cofactor of several antioxidant enzymes as GR and GPx [19]. In the present study, the increment in GSSG/GSH ratio in kidneys was found after CCM treatment. The alteration in the activities of these enzymes, together with the alteration in the GSH/GSSG system, reflects that CCM can induce alterations to the oxidative system in the kidney. These differences may be related to the sex hormones which play an important role in acute kidney failure. For example, there is a gender difference with regard to the severity of hepatic oxidative stress in acute uremia, with female rats displaying significant protection relative to male rats [20]. Moreover, the toxicity of doxorubicin was much stronger in male rats; toxicity was diminished after castration, which is known to downregulate the expression of renal OAT1 in rats [21, 22]. In the future, models such as orchidectomized males and ovariectomized females will be used to shed light on the role of hormone in the nephrotoxicity induced by *C. militaris.*

BUN and creatinine are considered as the traditional indicators of kidney damage;however, no change was observed in BUN and creatinine levels in our study. kIM-1 is a more sensitive biomarker which outperforms traditional biomarkers of kidney injury [23]. In our study, the low dose of CCM induced a minimal but significant increase in kIM-1 transcript levels in male rats, even in the absence of histopathological indications. kIM-1 gene expression increased significantly beginning at 1 g/kg treatment of CCM, although the change appeared at the protein level only from 3 g/kg treatment. Gene expression appeared to be more sensitive than either the kIM-1 protein level or urine assay. In the female rats, tubular damage induced by CCM at the high dose was less frequent and less severe, but it was captured by kIM-1 gene expression analysis. In accordance with the previous report [24], kIM-1 is expected to represent an important genomic marker for the potential screening of nephrotoxicants. 

Cordycepin (3′-deoxyadenosine), a purine nucleoside derivative, is an active component of C. *militaris*. UPLC analysis showed that the content of cordycepin in *C. militaris* is 2.18 mg/g in our study. Sohn et al. reported that the epididymal weights of middle-aged rats were dose dependently increased by treatment with cordycepin. All cordycepin-treated groups showed well-arranged spermatogonia, densely packed cellular material, and increased numbers of mature spermatozoa in the seminiferous lumen [25]. These may explain the increase in testis weight in the 2 g/kg and 3 g/kg groups in our study. Consistently, *C. sinensis* can enhance libido and sexual performance as well as ameliorates impaired reproductive functions such as impotency or infertility in males [26]. On the other hand, total cholesterol was lower in CCM-treated groups (both genders) than in the control group, and similar decrease was observed in *C. guangdongensis*-treated rats [15]. Interestingly, it is reported that *C. sinensis* extract decreased the serum cholesterol level of rats fed with a cholesterol-enriched diet [27, 28]. Cordycepin prevents hyperlipidemia in hamsters fed a high-fat diet via activation of AMP-activated protein kinase [29, 30]. Therefore, the change on testis weight and total cholesterol may be regarded as a positive effect.

## 5. Conclusions

For the first time, our results clearly indicate that CCM can induce nephrotoxicity to rats and male rats have greater sensitivity to nephrotoxicity than the female rats. Although the toxic dose level was relatively high, it is enough to arouse the public attention on the safety of herbal medicine. One of the oxidative stress markers such as GSSG/GSH ratio was increased due to CCM treatment. CCM also decreased the activity of antioxidant enzymes as well as promoted the protein and mRNA levels of KIM-1. Herein, we conclude that the mechanism may be explained, partially, by depleting the antioxidant defense system.

## Figures and Tables

**Table 1:** Calibration curves and contents of nucleosides and nucleobases in CCM.

	Calibration range (*μ*g/mL)	*r* ^2^	Contents, mg/g
Uridine	4.8–240	1	1.41
Adenine	0.72–36	0.9999	0.05836
Guanosine	2.56–128	1	0.45
Adenosine	2.976–148.8	1	0.86
Cordycepin	6.8–340	1	2.18

**Table 2:** Sequences of the primers.

Gene	GenBank number	Forward	Reverse
Beta-Actin	V01217	TGACCGAGCGTGGCTACAG	GGGCAACATAGCACAGCTTCT
KIM-1	NM-173149	TGGCACTGTGACATCCTCAGA	GCAACGGACATGCCAACATA

**Table 3:** Weekly body weight (g) of rats treated with CCM for 28 days.

Treatment (g/kg)	Days				
	0	7	14	21	28
Male rats					
0	81.1 ± 4.4	147.1 ± 8.6	224.2 ± 26.1	290.2 ± 26.1	375.1 ± 39.7
1	81.2 ± 4.3	142.7 ± 9.2	213.6 ± 25.1	277.3 ± 20.2	359.8 ± 32.2
2	82.4 ± 4.1	139.5 ± 10.5	201.2 ± 28.1	253.4 ± 26.3	313.7 ± 35.4
3	82.1 ± 4.2	133.2 ± 7.2	179.5 ± 11.8	248.5 ± 21.4	319.6 ± 31.4
Female rats					
0	80.1 ± 4.4	135.6 ± 17.5	187.4 ± 10.2	211.5 ± 10.1	247.2 ± 25.1
1	80.2 ± 4.2	129.6 ± 19.6	174.4 ± 10.3	199.7 ± 11.2	237.6 ± 24.2
2	80.8 ± 4.2	126.8 ± 14.7	172.6 ± 16.6	193.6 ± 17.5	217.5 ± 28.3
3	80.3 ± 4.1	131.9 ± 17.3	171.6 ± 16.7	191.5 ± 20.5	224.5 ± 24.4

Each value is expressed as mean ± SD (*n* = 10/sex/dose).

**Table 4:** Organ-to-body weight ratios of rats treated with CCM for 28 days.

Treatment (g/kg)	Liver (%)	Spleen (%)	Kidney (%)	Testis (%)	Ovary (%)
Male rats					
0	3.44 ± 0.19	0.25 ± 0.03	0.86 ± 0.02	0.88 ± 0.07	
1	3.39 ± 0.19	0.24 ± 0.03	0.89 ± 0.07	0.89 ± 0.09	
2	3.33 ± 0.22	0.24 ± 0.02	0.94 ± 0.11	1.03 ± 0.09**	
3	3.37 ± 0.12	0.24 ± 0.02	0.91 ± 0.06*	1.04 ± 0.09**	
Female rats					
0	3.52 ± 0.45	0.23 ± 0.04	0.80 ± 0.13		0.06 ± 0.01
1	3.46 ± 0.34	0.22 ± 0.02	0.84 ± 0.11		0.05 ± 0.01
2	3.36 ± 0.16	0.22 ± 0.04	0.73 ± 0.05		0.06 ± 0.01
3	3.55 ± 0.17	0.22 ± 0.04	0.77 ± 0.06		0.06 ± 0.01

Each value is expressed as mean ± SD (*n* = 10/sex/dose).

*P < 0.05;  **P < 0.01.

**Table 5:** Hematological values and biochemical parameters of rats treated with CCM for 28 days.

Item	Treatment (g/kg)			
	0	1	2	3
Male rats				
WBC (×10^9^/L)	7.1 ± 1.5	6.9 ± 1.6	6.3 ± 1.1	6.9 ± 1.7
RBC (×10^12^/L)	7.1 ± 1.3	7.3 ± 1.2	7.3 ± 1.4	7.3 ± 1.4
Hemoglobin (g/L)	149.2 ± 15.1	148.3 ± 15.2	147.4 ± 19.1	147.5 ± 16.3
Lymphocyte (%)	78.8 ± 11.9	78.5 ± 12.5	76.8 ± 13.5	78.9 ± 12.5
Neutrophil (%)	13.4 ± 3.6	14.1 ± 3.5	15.3 ± 7.0	14.9 ± 3.9
Monocyte (%)	6.7 ± 2.3	6.2 ± 1.5	6.8 ± 2.0	6.6 ± 2.5
Eosinophil (%)	0.7 ± 0.4	0.8 ± 0.4	0.7 ± 0.5	0.8 ± 0.4
Basophil (%)	0.4 ± 0.2	0.4 ± 0.2	0.4 ± 0.2	0.4 ± 0.3
ALT (U/L)	22 ± 3	22 ± 3	27 ± 5*	29 ± 5**
AST (U/L)	97 ± 8	109 ± 23	125 ± 13**	129 ± 15**
Totol protein (g/L)	52.1 ± 3.3	52.3 ± 4.6	52.4 ± 3.8	55.1 ± 3.7
Albumin (g/L)	30.0 ± 0.9	30.0 ± 1.3	30.5 ± 0.9	30.1 ± 0.8
BUN (mg/dL)	11.9 ± 2.2	12.2 ± 2.1	12.1 ± 3.3	12.5 ± 4.6
Creatinine (mg/dL)	0.55 ± 0.11	0.54 ± 0.16	0.56 ± 0.17	0.54 ± 0.15
TC (mmol/L)	1.79 ± 0.30	1.66 ± 0.20	1.56 ± 0.17	1.43 ± 0.29*
Triglyceride (mmol/L)	0.72 ± 0.33	0.65 ± 0.19	0.50 ± 0.07	0.52 ± 0.17
Glucose (mmol/L)	6.02 ± 0.40	6.09 ± 0.89	5.91 ± 0.98	5.93 ± 0.78
Na (mmol/L)	137 ± 1	138 ± 1	138 ± 2	139 ± 2
K (mmol/L)	5.10 ± 0.65	5.22 ± 0.56	5.15 ± 0.78	5.14 ± 0.54
Cl (mmol/L)	98 ± 1	97 ± 1	97 ± 2	98 ± 3
Female rats				
WBC (×10^9^/L)	7.5 ± 1.4	5.9 ± 1.1	6.0 ± 1.1	6.2 ± 1.4
RBC (×10^12^/L)	7.4 ± 0.3	7.7 ± 0.4	7.7 ± 0.4	7.5 ± 0.5
Hemoglobin (g/L)	151.2 ± 6.1	153.3 ± 10.4	151.5 ± 6.1	150.4 ± 6.2
Lymphocyte (%)	79.1 ± 14.0	77.5 ± 11.2	80.1 ± 14.9	78.8 ± 13.7
Neutrophil (%)	13.9 ± 3.5	14.0 ± 5.7	13.3 ± 4.9	13.3 ± 3.5
Monocyte (%)	5.7 ± 1.7	7.6 ± 3.3	5.9 ± 1.4	6.8 ± 2.8
Eosinophil (%)	0.8 ± 0.7	0.5 ± 0.5	0.4 ± 0.4	0.8 ± 0.5
Basophil (%)	0.4 ± 0.2	0.3 ± 0.2	0.3 ± 0.2	0.4 ± 0.2
ALT (U/L)	20 ± 3	22 ± 3	25 ± 4*	28 ± 4**
AST (U/L)	92 ± 9	98 ± 11	112 ± 14**	126 ± 12**
Totol protein (g/L)	56.0 ± 4.6	56.9 ± 2.8	56.8 ± 2.1	53.0 ± 1.9
Albumin (g/L)	34.4 ± 2.5	33.2 ± 1.5	33.1 ± 0.9	30.6 ± 0.9
BUN (mg/dL)	12.5 ± 2.1	12.8 ± 3.4	13.6 ± 3.9	13.2 ± 3.1
Creatinine (mg/dL)	0.57 ± 0.05	0.60 ± 0.11	0.63 ± 0.09	0.60 ± 0.11
TC (mmol/L)	1.97 ± 0.23	1.77 ± 0.26	1.89 ± 0.32	1.56 ± 0.21*
Triglyceride (mmol/L)	0.67 ± 0.37	0.59 ± 0.36	0.42 ± 0.12	0.50 ± 0.25
Glucose (mmol/L)	5.98 ± 0.99	5.88 ± 1.00	5.72 ± 0.70	5.80 ± 0.87
Na (mmol/L)	143 ± 1	142 ± 2	143 ± 1	139 ± 1
K (mmol/L)	5.65 ± 0.72	5.63 ± 0.83	5.62 ± 0.51	5.60 ± 0.49
Cl (mmol/L)	100 ± 2	98 ± 2	98 ± 2	96 ± 2

Each value is expressed as mean ± SD (*n* = 10/sex/dose). RBC: red blood cell count; WBC: white blood cell count; AST: aspartate aminotransferase; ALT: alanine aminotransferase; BUN: blood urea nitrogen; TC: total cholesterol; Cl: chloride; Na: sodium; K: potassium.

*P < 0.05;  **P < 0.01.

**Table 6:** Incidence of renal histopathological findings of rats treated orally with CCM for 28 days from weaning.

		Treatment (g/kg)							
Findings	Grade	Male rats				Female rats			
		0	1	2	3	0	1	2	3
Glomerular exudate	++	0	0	1	3	0	0	0	0
	+++	0	0	3	7	0	0	0	0
Degeneration, tubular epithelium	±	1	0	0	0	0	0	0	1
	+	2	0	0	0	1	0	0	1
	++	0	0	2	5	0	0	0	1
	+++	0	0	2	4	0	0	0	0
Necrosis, tubular epithelium	+	0	0	0	2	0	0	0	0
	++	0	0	1	3	0	0	0	0
	+++	0	0	0	1	0	0	0	0
Nuclear condensation, tubular epithelium	+	0	0	0	4	0	0	0	0
	++	0	0	3	6	0	0	0	0
Inflammation, interstitial	±	0	0	0	0	0	0	0	1
	+	0	0	0	1	0	0	0	0

(±): very slight; (+): slight; (++): moderate; (+++): severe.

**Table 7:** Effects of CCM exposure on antioxidant enzyme activities in kidneys of ablactated rats.

Item	Treatment (g/kg)			
	0	1	2	3
Male rats				
SOD (U/mg protein)	29.4 ± 6.4	29.3 ± 5.2	26.9 ± 6.2	23.2 ± 5.5*
CAT (*μ*mol/min/mg protein)	154.1 ± 21.5	150.2 ± 23.7	149.3 ± 24.7	126.1 ± 22.9*
GR (mU/mg protein)	552.1 ± 101.3	550.3 ± 110.6	525.4 ± 112.8	520.2 ± 119.9
GPx (mU/mg protein)	923.6 ± 213.5	880.5 ± 187.9	856.3 ± 198.3	840.9 ± 201.5*
GST (mU/mg protein)	0.68 ± 0.15	0.68 ± 0.16	0.69 ± 0.17	0.65 ± 0.18
GSSG/GSH ratio	0.65 ± 0.11	0.64 ± 0.16	0.78 ± 0.12	0.97 ± 0.25*
Female rats				
SOD (U/mg protein)	35.2 ± 7.1	34.1 ± 6.4	31.2 ± 6.5	28.7 ± 4.4*
CAT (*μ*mol/min/mg protein)	143.2 ± 18.3	145.2 ± 21.8	140.5 ± 23.5	136.3 ± 21.3
GR (mU/mg protein)	514.5 ± 121.7	502.2 ± 104.9	497.3 ± 112.2	505.3 ± 124.5
GPx (mU/mg protein)	822.5 ± 194.4	823.4 ± 199.2	801.5 ± 195.7	811.8 ± 215.7
GST (mU/mg protein)	0.54 ± 0.11	0.55 ± 0.12	0.58 ± 0.13	0.54 ± 0.12
GSSG/GSH ratio	0.71 ± 0.10	0.73 ± 0.15	0.79 ± 0.17	0.96 ± 0.21*

Each value is expressed as mean ± SD (*n* = 10/sex/dose).

SOD: superoxide dismutase; CAT: catalase; GR: glutathione reductase; GPx: glutathione peroxidase; GST: glutathione S-transferase; GSSG: glutathione oxidized form; GSH: glutathione reduced form.

*P < 0.05.

## Abbreviations

AST: Aspartate aminotransferase
ALT: Alanine aminotransferase
BUN: Blood urea nitrogen
CAT: Catalase
CCM: Cordyceps militaris
Cl: Chloride
GPx: Glutathione peroxidase
GR: Glutathione reductase
GSSG: Glutathione oxidized form
GSH: Glutathione reduced form
GST: Glutathione S-transferase
K: Potassium;
KIM-1: Kidney injury molecule-1
Na: Sodium
RBC: Red blood cell count
SOD: Superoxide dismutase
TC: Total cholesterol
WBC: White blood cell count.
